# Pharmacological activation of BK channels protects against LPS-induced pneumonia

**DOI:** 10.1038/s41598-025-08902-6

**Published:** 2025-08-19

**Authors:** Tatiana Zyrianova, Benjamin Lopez, Janelle SooHoo, Christian Boehmer, Annie Ye, Hailey Kang, Nairrita Majumder, Andreas Schwingshackl

**Affiliations:** Department of Pediatrics, University of California Los Angeles, Los Angeles, CA 90095 USA

**Keywords:** Cell biology, Physiology, Medical research, Molecular medicine, Pathogenesis

## Abstract

Bacterial pneumonia causes 1.4 million deaths annually worldwide. Besides antibiotics, current treatments are mostly supportive, and no other targeted therapies exist that improve patient outcomes. Key features of bacterial pneumonia include alveolar inflammation, including inflammatory cell infiltration, mediator release, and alveolar-capillary barrier dysfunction. We previously demonstrated that plasma membrane hyperpolarization via large conductance K^+^ (BK) channels reduces pro-inflammatory mediator release from TNF-α- or lipopolysaccharide (LPS)-treated pulmonary endothelial cells. Building on those findings, this study evaluates pharmacological BK channel activation as a potential treatment for LPS-induced pneumonia in a mouse model and explores its molecular mechanisms. We found that BK channel activation with NS1619 in LPS-infected mice reduced broncho-alveolar lavage fluid total cell and neutrophil counts, CCL-2 concentrations, and ROS and H_2_O_2_ production, and increased antioxidant superoxide dismutase and catalase levels. These effects were not linked to glutathione, neutrophil myeloperoxidase, elastase, or extracellular traps. These protective effects were replicated with a structurally different BK channel activator, NS19504. At the cellular level, both NS1619 and NS19504 reduced LPS-induced ROS production in primary human alveolar epithelial cells, whereas LPS had no effect on endothelial ROS production. Our findings suggest that pharmacological BK channel activation could serve as a new therapeutic target against bacterial pneumonia.

## Introduction

Globally, bacterial pneumonia constitutes one of the most devastating public health issues owing to its widespread incidence, disease severity, and associated high morbidity and mortality rates in people of all ages, in addition to substantial medical and economic costs. Worldwide, each year bacterial pneumonia causes 1.4 million fatalities among adults and more than 800,000 among children under the age of 5 years^1^. Gram-negative organisms are frequent etiologies of bacterial pneumonia and are associated with up to 60% mortality^2^. Therapeutic options are limited to antibiotics and supportive therapies such as supplemental oxygen, but no molecular targets are known that translate into improved patient outcomes^3^. Unfortunately, although antibiotic therapies and oxygen supplementation can be lifesaving interventions in critical cases, these therapies are also associated with significant adverse effects and can promote further lung injury and multiorgan failure^4^.

In search for new therapies, prior studies from our group suggest that electrical plasma membrane potential (Em) hyperpolarization via pharmacological activation of lung K^+^ channels may represent a novel anti-inflammatory approach^5–8^. In earlier studies, we found that so-called 2-pore domain K^+^ channels of the TREK subfamily play a regulatory role in hyperoxia-, mechanical stretch- and TNF-a-induced alveolar epithelial cell injury^4,6–10^. Additionally, we showed that pharmacological activation of TREK channels protects mice against hyperoxia- and Influenza A virus-induced acute lung injury^11^. More recently, we discovered that activation of Ca^2+^-activated large conductance K^+^ (BK) channels, which are characterized by a much larger single cell conductance (~ 100–300 pS) than TREK channels^12^, similarly causes Em hyperpolarization, particularly in pulmonary endothelial cells. Specifically, these previous in vitro studies revealed that pharmacological BK channel activation with two structurally-different compounds, NS1619 and NS19504, decreases lipopolysaccharide (LPS)- or tumor necrosis factor (TNF)-α-induced pro-inflammatory CCL-2 release from human pulmonary endothelial cells through inhibition of Ca^2+^-dependent MAPK pathways^5,13^. However, whether these promising BK channel-mediated protective effects seen in isolated cell cultures can be translated into in vivo pneumonia models, remains unknown. Therefore, this study was designed to determine whether direct intra-tracheal administration of the BK channel activating compounds NS1619 and NS19504 can protect mice against LPS-induced pneumonia, and identify the molecular pathways underlying BK channel activation-mediated protection.

## Results

### Pharmacological BK channel activation reduces LPS-induced pneumonia in mice

**Fig. 1:**
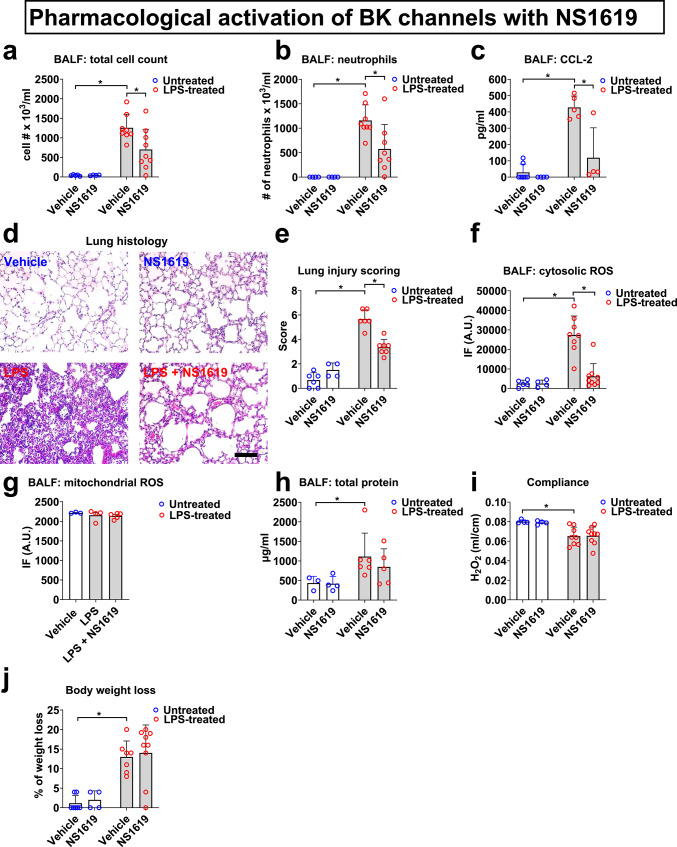

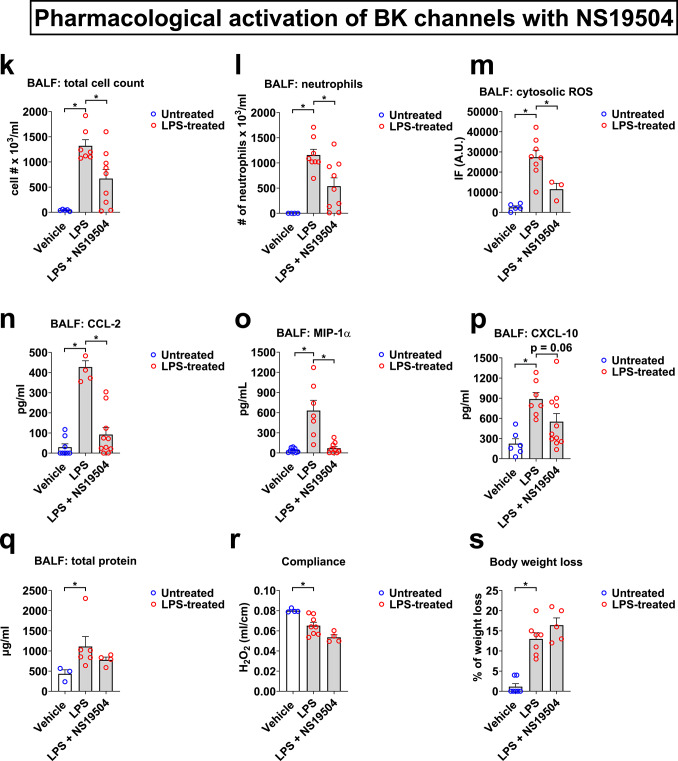
Pharmacological BK channel activation protects mice against LPS-induced pneumonia. LPS infection (*i.t.*, 10 mg/kg) increased BALF total cell (**a**, **k**) and neutrophil counts (**b**, **l**), and BALF CCL-2 (**c**, **n**), MIP-1α (**o**) and CXCL-10 (**p**) concentrations, lung injury scores (**d**, **e**), and cytosolic ROS production by BALF cells (**f**, **m**). Two doses of the BK activator NS1619 or NS19504 (*i.t.*, 0.66 mg/kg), given at 0 and 24 h improved all measured markers of acute lung injury. In contrast, mitochondrial ROS production (**g**), BALF total protein levels (**h**, **q**), quasi-static lung compliance (**i**, **r**), and body weight loss (**j**, **s**) were not affected by NS1619 treatment. Control mice received equimolar drug vehicle injections. n = 3–10 mice per group; individual experimental data points are depicted within each bar; bars depict mean ± SEM; *p < 0.05; IF—intensity of fluorescence; A.U.—arbitrary units; scale bar: 650 µm.

Intratracheal (*i.t.*) LPS administration is a well-established approach to model for Gram negative pneumonia^14^. Our findings demonstrate that LPS (10 mg/kg) significantly increases total cell counts in bronchoalveolar lavage fluid (BALF; Fig. 1a, k), which is almost exclusively composed of infiltrating neutrophils (over 92% of BALF cells) and macrophages (approx. 8% of BALF cells; Fig. 1b, l). This immune/inflammatory cell influx is associated with a significant increase in CCL-2 levels in BALF, a well-established neutrophil/macrophage chemoattractant (Fig. 1c, n). In addition to immune/inflammatory cell infiltration, we also found an increase in cytosolic but not mitochondrial ROS preproduction by BALF cells in LPS-treated mice (Fig. 1d–g, m). Histological lung tissue examination and lung injury scoring (LIS) on H&E-stained tissue sections showed significant inflammation after 48 h of LPS infection. Representative images and a composite histological LIS are shown in Fig. 1d and e. *I.t.* administration of the BK channel activator NS1619 (0.66 mg/kg) at 0 and 24 h following LPS infection reversed LPS-induced total BALF cell counts and neutrophil infiltration into the alveolar space, CCL-2 secretion, cytosolic ROS production by BALF cells, and LIS parameters (Fig. 1a–f). In a separate set of experiments, we confirmed the protective effects of pharmacological BK channel activation against LPS-induced pneumonia with another, structurally different BK activator, NS19504^15^. *I.t.* administration of NS19504 (1.33 mg/kg) at 0 and 24 h following LPS infection reversed not only LPS-induced total BALF cell counts and neutrophil infiltration, CCL-2 secretion, and cytosolic ROS production but also MIP-1α cytokine secretion (Fig. 1k–o), while inhibition of CXCL-10 secretion was slightly short of meeting statistical significance criteria (*p* = 0.06; Fig. 1p).

Of note, initial dose–response experiments with NS1619 (0.66 mg/kg and 1.33 mg/kg) showed higher efficiency in reducing inflammatory parameters with the lower than the higher dose (data not shown). Therefore, we chose the 0.66 mg/kg dose for the rest of the study. In contrast, we observed a more efficient response with the higher 1.33 mg/kg dose of the NS19504 activator compared to NS1619 (data not shown). Additionally, since NS1619 alone did not affect any of the reported endpoints in non-infected mice (Fig. 1a–j), to reduce animal numbers, we proceeded with evaluating NS19504 responses only in LPS-infected mice (Fig. 1k–s).

LPS treatment also resulted in worsening of additional lung injury markers, including an increase in BALF total protein concentrations (from 433 ± 100 to 1112 ± 245 g/ml; Fig. 1h, q), decrease in quasi-static lung compliance (from 0.08 ± 0.0009 to 0.06 ± 0.003 ml/cm of H_2_O; Fig. 1i, r), and increase in body weight loss of 13 ± 0.6% (Fig. 1j and s), but neither NS1619 or NS19504 affected these markers of illness. Altogether, these findings suggest that *i.t.* BK channel activation results in significant anti-inflammatory effects in the alveolar compartment, as evidenced by improvements in LPS-induced immune/inflammatory cell infiltration, alveolar CCL-2 and MIP-1α chemokine secretion, and ROS production by BALF cells, but has no significant effect on alveolar-capillary barrier function since BALF total protein levels remained elevated.

**Fig. 2:**
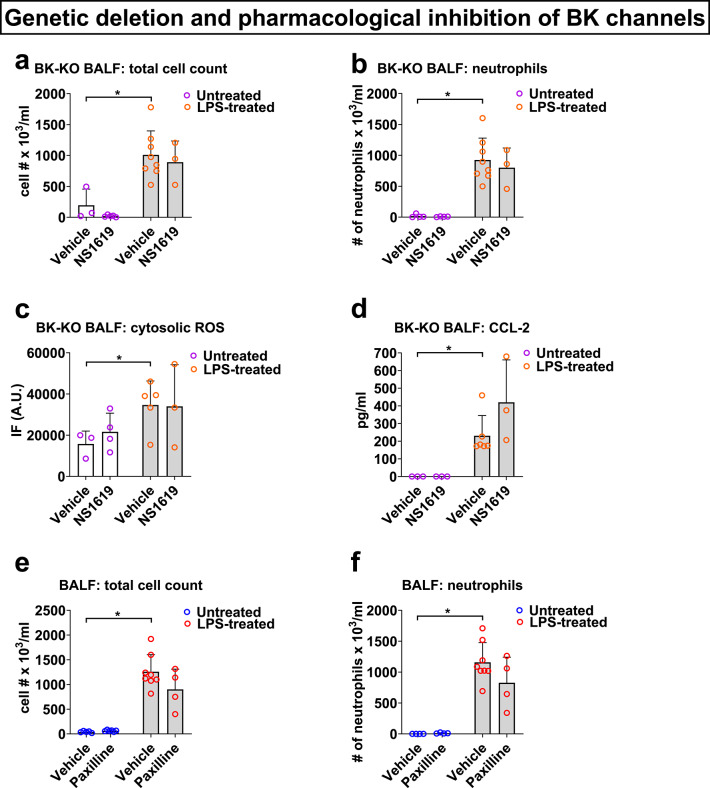
NS1619 targets BK channels: LPS-infected (*i.t.*, 10 mg/kg) BK-KO mice lack the protective effects of NS1619 (*i.t.*, 0.66 mg/kg, given in 2 doses at 0 and 24 h) on BALF total cell (**a**) and neutrophil counts (**b**), cytosolic ROS production by BALF cells (**c**), and BALF CCL-2 (**d**) concentrations. Pharmacological BK channel inhibition has no effects on BALF total cell (**e**) and neutrophil (**f**) counts, indicating that BK channels are mostly closed at baseline and are thus amendable to pharmacological activation. WT mice were infected with LPS (*i.t.*, 10 mg/kg) with or without the BK channel blocker Paxilline (*i.t.*, 1.33 mg/kg), given in 2 doses at 0 and 24 h. Control mice received equimolar drug vehicle injections; n = 3–8 mice per group; individual experimental data points are depicted within each bar; bars depict mean ± SEM; *p < 0.05; IF—intensity of fluorescence; A.U.—arbitrary units.

To validate that the observed protective effects of NS1619 and NS19504 indeed occurred via BK channel activation-mediated effects, for proof-of-concept we repeated these experiments in global BK-KO mice, and no longer observed a reduction in total and neutrophil BALF cell counts, CCL-2 secretion, or cytosolic ROS production by BALF cells in LPS-infected mice (Fig. 2a–d). Furthermore, similar to genetic BK channel depletion, pharmacological inhibition of BK channels with *i.t.* Paxilline (1.33 mg/kg)^16^, administered at 0 and 24 h after LPS treatment, also lacked any effects on total BALF inflammatory cell or neutrophil infiltration (Fig. 2e, f).

### Pharmacological BK channel activation reduces LPS-induced alveolar oxidative stress in BALF cells

**Fig. 3:**
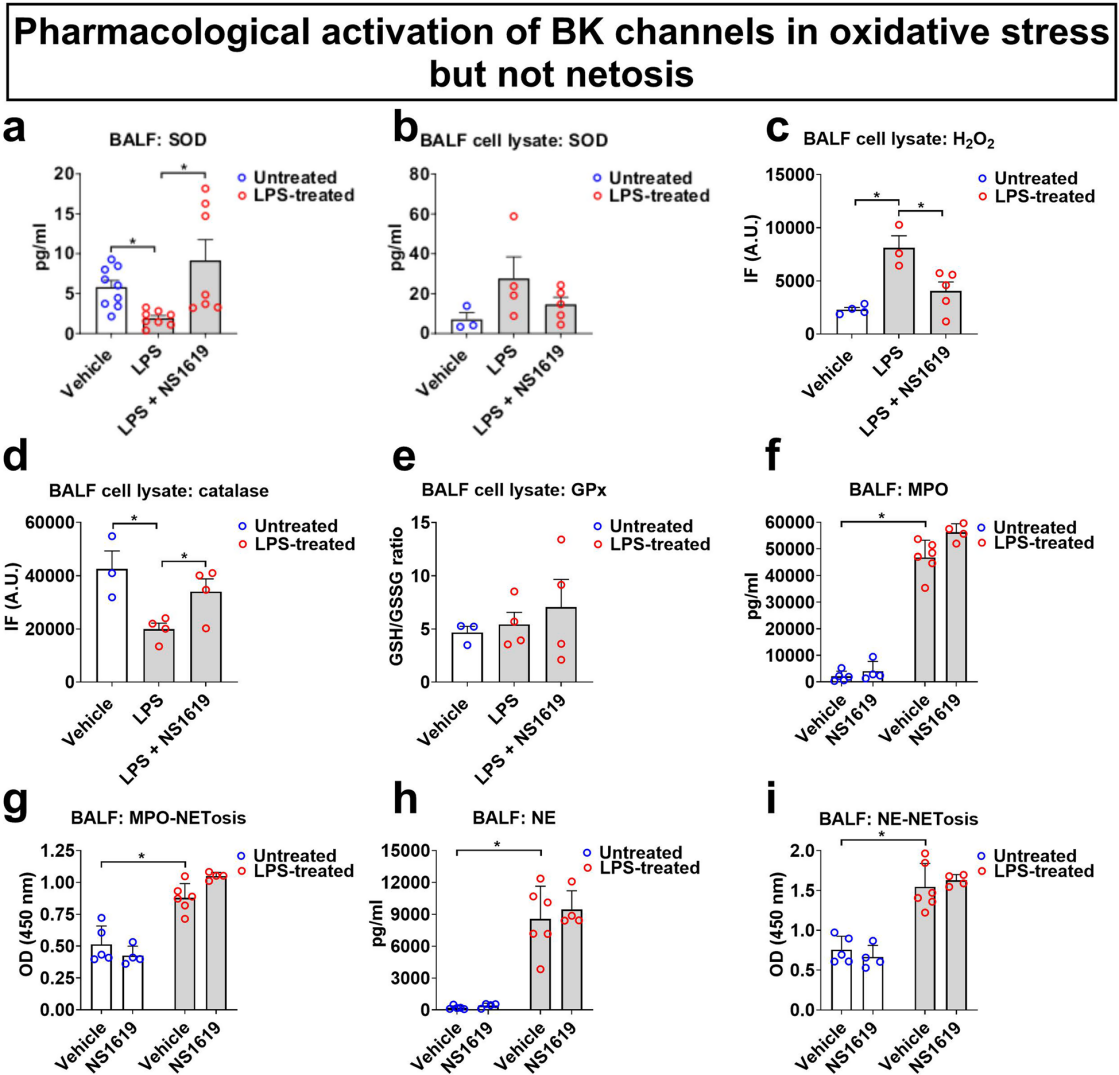
Pharmacological BK channel activation promotes antioxidant effects in BALF but does not affect NETosis. WT mice were infected with LPS (*i.t.*, 10 mg/kg) with or without the BK channel activator NS1619 (*i.t.*, 0.66 mg/kg) given in 2 doses at 0 and 24 h. Control mice received equimolar drug vehicle injections. LPS infection decreased BALF SOD (**a**) and catalase (**d**) levels, and increased H_2_O_2_ levels (**c**). NS1619 treatment reversed these effects. Neither LPS or LPS + NS1619 treatment affected BALF cell lysate SOD levels (**b**). GSH/GSSH ratios, which reflect GPx activity, were not altered by LPS or NS1619 (**e**); LPS induced neutrophil myeloperoxidase (MPO; **f**) and neutrophil elastase (NE; **h**) secretion, and neutrophil extracellular traps formation (NETosis; **g**; **i**), which were not affected by NS1619 treatment. n = 3–9 mice per group; individual experimental data points are depicted within each bar; bars depict mean ± SEM; *p < 0.05; OD—optical density.

To further dissect the role of BK channels in the regulation of ROS production by BALF cells, we measured the effects of NS1619 on several antioxidant mechanisms in BALF supernatants and BALF cell lysates (Fig. 3). Briefly, superoxide dismutase (SOD) converts superoxide anions to H_2_O_2_, while catalase and glutathione peroxidase (GPx) convert H_2_O_2_ to water. GPx reductase replenishes reduced glutathione (GSH) pools from oxidized glutathione (GSSG) using NADPH as reducing equivalents^17,18^. Our results demonstrate a reduction in SOD activity levels in BALF supernatants from LPS-infected mice when compared to non-infected controls. Activation of BK channels with NS1619 restored these SOD activity levels back to baseline (Fig. 3a). In contrast to secreted SOD, SOD levels in BALF cell lysate were not affected by LPS or NS1619 treatment (Fig. 3b). H_2_O_2_ levels were increased in BALF cell lysates from LPS-infected mice, and this effect was reversed by NS1619 treatment (Fig. 3c). Catalase levels, the enzyme that uses H_2_O_2_ as a substrate^18^, were decreased in BALF cell lysates of LPS-infected mice, and this effect was also counteracted by NS1619 (Fig. 3d). GSH/GSSH ratios in BALF cell lysates, an indicator of GPx activity^18^, were not affected by LPS infection or NS1619 treatment (Fig. 3e). Altogether, these results suggest that the observed protective effects against LPS-induced oxidative stress in the alveolar compartment are due to restoration of SOD and catalase levels, but not GPx activity.

To further explore the anti-inflammatory and protective potential of BK activation with NS1619 and given the strong neutrophil predominance in LPS-infected mice, we measured the concentrations of two well-established markers of neutrophil activation in BALF, myeloperoxidase (MPO) and neutrophil elastase (NE)^19,20^. Both MPO and NE release from BALF cells was higher in LPS-infected mice when compared to their non-infected controls (Fig. 3f, h), but pharmacological BK activation with NS1619 did not inhibit MPO or NE levels. Furthermore, NETosis, another marker of neutrophil activation and a defense strategy against microbial pathogens^21^, was quantified using MPO-specific or NE-specific capture antibodies, followed by MPO-DNA or NE-DNA antibody detection. NETosis was increased in BALF cells from LPS-treated mice, but this effect was not inhibited by NS1619 (Fig. 3g, i).

These results collectively suggest that the observed protective effects of BK channel activation against LPS-induced oxidative stress in the alveolar compartment are predominantly due to inhibition of chemotactic CCL-2 and MIP-1α secretion, inhibition of oxidative stress, and enhancement of anti-oxidant responses.

### Pharmacological BK channel activation reduces ROS production in primary human alveolar epithelial cells

**Fig. 4:**
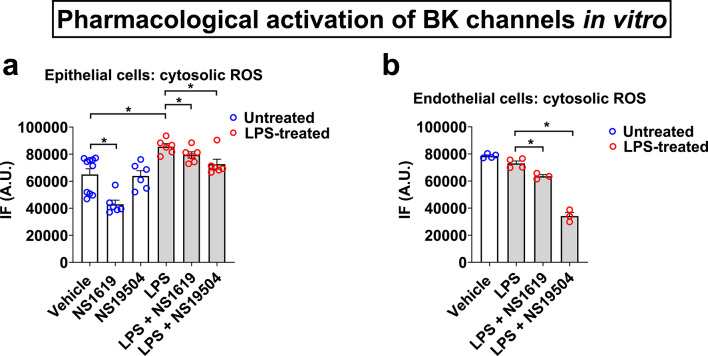
Pharmacological activation of BK channels with NS1619 (30 μM) or NS19504 (30 μM) administered at 0 h protects primary human alveolar epithelial cells (HPAEpiC against LPS (2 μg/ml) -induced ROS production (**a**). In contrast, primary human pulmonary artery endothelial cells (HPAEC) showed no ROS response to LPS treatment (**b**). Control cells were treated with an equimolar drug vehicle. n = 3–7 separate experimental repeats; individual experimental data points are shown within each bar; bars represent mean ± SEM; *p < 0.05; IF – intensity of fluorescence; A.U.—arbitrary units.

To further investigate the role of BK channels in regulating cytosolic ROS production across different alveolar cell types, we established an in vitro model of LPS-induced inflammation by infecting primary human alveolar epithelial cells (HPAEpiC) and primary human pulmonary artery endothelial (HPAEC) cells with LPS (2 µg/ml). In epithelial cells, LPS treatment increased ROS production, which peaked after 11 h and was reduced by pharmacological activation of BK channels with either NS1619 (30 µM) or NS19504 (30 µM). Notably, baseline ROS production was only affected by NS1619 but not NS19504 (Fig. 4a). In contrast to epithelial cells, in endothelial cells LPS did not induce ROS production, but NS1619 and NS19504 appeared to decrease baseline ROS levels (Fig. 4b).

Altogether, these findings suggest that BK-mediated inhibition of LPS-induced ROS production in the alveolar compartment is primarily due to anti-oxidant effects on immune/inflammatory cells in the BALF (composed of neutrophils and macrophages) and alveolar epithelial cells, but not endothelial cells.

### BK channel-mediated network analysis reveals cytokine and ROS clusters connected through H_2_O_2_ in an in silico model

**Fig. 5:**
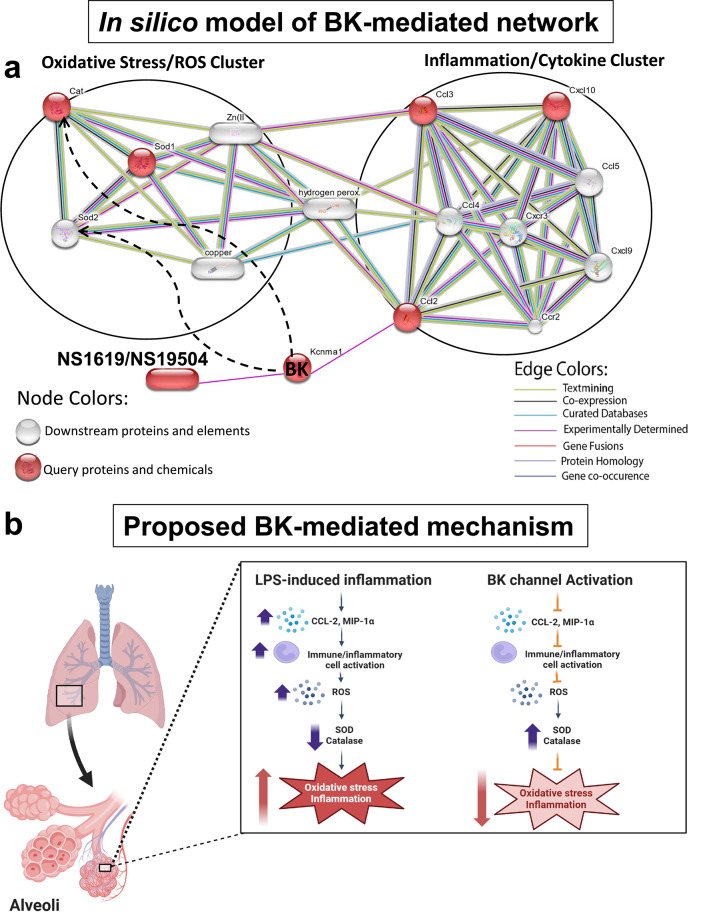
(**a**) BK channel-mediated network analysis predicts cytokine and ROS clusters connected through hydrogen peroxide (H_2_O_2_) in an in silico model. A protein-drug interaction network was constructed using the STITCH database to investigate the relationships between BK channels, oxidative stress, and inflammatory molecules in an LPS-induced pneumonia model. The network includes BK channels (KCNMA1), BK channel activators (NS1619/NS19504), neutrophil/macrophage chemoattractants (CCL-2, MIP-1α, CXCL-10), and oxidative stress enzymes (SOD1, catalase). The network analysis identified two distinct clusters: a ROS cluster (including SOD2, zinc, and copper) and a cytokine cluster (including CCL-4, CXCR-2, CCR-2, CCL-5, and CXCL-9). These clusters are interconnected by H_2_O_2_, linking ROS production and cytokine release to BK channels in LPS-induced pneumonia. Dashed lines represent connections identified in this study; red nodes indicate query proteins obtained in this study, while gray nodes represent the most likely downstream interaction partners with a high confidence level (0.95 on a 0–1 scale). (**b**) Schematic diagram of the proposed mechanism underlying BK channel activation-mediated protection against LPS-induced oxidative stress.

To further investigate the relationships between BK channels, oxidative stress, and inflammatory molecules in our LPS-induced pneumonia model, we constructed a protein-drug interaction network using the STITCH database and integrating the following components: (i) BK channels (KCNMA1, the pore-forming α-subunit of the channel), (ii) the BK channel activating drugs NS1619/NS19504, (iii) the neutrophil/macrophage chemoattractants CCL-2, MIP-1α (CCL-3), and CXCL-10 cytokines, (iv) SOD1, and (v) catalase (Cat) (Fig. 5a). This predictive protein-drug interaction model identified the top nine interaction partners within our network, which fell into two distinct clusters: oxidative stress (ROS cluster) and inflammation (cytokine cluster). The ROS cluster includes the SOD2 enzyme and two elements, zinc, and copper, while the cytokine cluster consists of CCL-4, CXCR-2, CCR-2, CCL-5, and CXCL-9 proteins. These two clusters are interconnected through hydrogen peroxide (H_2_O_2_), which bridges the two distinct inflammatory processes revealed in the clusters: the production of ROS and cytokines (Fig. 5a). Collectively, our in silico model predicts a previously unknown mechanism in LPS-induced pneumonia by which pharmacological BK activation with NS1619/NS19504 chemicals regulates ROS production and cytokine release via the intermediary molecule, H_2_O_2._

## Discussion

Treating pneumonia caused by gram-negative bacteria remains challenging due to high mortality rates exceeding 60% and significant antibiotic resistance^2^. Current treatments primarily include antimicrobial therapy and oxygen supplementation, but no targeted anti-inflammatory interventions exist to improve patient outcomes^2^. Pathophysiologically, bacterial pneumonia is characterized by alveolar inflammation, consisting of immune/inflammatory cell infiltration and alveolar-capillary barrier dysfunction^22^. In acute lung injury models, neutrophils and macrophages are the predominant immune/inflammatory cell type found in inflamed alveolar spaces response, and together with resident alveolar epithelial and endothelial cells create a proinflammatory environment through cytokine secretion and ROS production^4,11,14,23,24^.

In search for new anti-inflammatory strategies, we previously found that pharmacological activation of BK channels reduces inflammatory mediator release from TNF-α- and LPS-stimulated human pulmonary endothelial cells, likely through plasma membrane hyperpolarization^5,13^. In this study, we tested whether these in vitro findings could be translated into an in vivo model. In a bacterial pneumonia model, we found that two structurally different BK activators, NS1619 and NS19504^15^ protect mice against LPS-induced immune/inflammatory cell infiltration and ROS production by BALF cells. These effects appear to be mediated by reducing the secretion of the neutrophil/macrophage chemoattractants CCL-2^25^ and MIP-1α^26^ and upregulating antioxidant responses, suggesting BK channels as potential targets for anti-inflammatory drug design against bacterial pneumonia. Notably, CCL-2 and MIP-1α are elevated in the lungs of bacterial pneumonia patients^26,27^, and our own studies showed that NS1619 reduced the levels of these two cytokines in both mice and human lung endothelial cells^5,13^. Given that these cytokines are secreted by various lung cells, including alveolar epithelial cells, neutrophils, and macrophages^11,14,28^, the effect of NS1619/NS19504 on CCL-2 and MIP-1α levels and neutrophil/macrophage accumulation and activation suggests that these BK activators target multiple cell types in the lung.

NS1619 is a well-established BK channel activating compound^29^, but since we employed it for the first time in a mouse pneumonia model, it was important to confirm that the observed protective effects stem, indeed, from BK channel activation and not potential off-target effects. To address this question, we repeated key experiments with another structurally different BK activator, NS19504^15^ (Fig. 1). Together with the lack of protective effects of NS1619 in BK-KO mice (Fig. 2), these findings provide us with a high confidence level that the observed anti-inflammatory effects are indeed BK channel-specific. The additional studies using the established BK channel blocker Paxilline provide further interesting insights into BK channel physiology. The lack of effect of Paxilline on BALF total and neutrophil cell counts (Fig. 2), combined with the protective effects of the BK channel activator NS1619 suggest that (i) at baseline most BK channels are closed and (ii) BK channels are readily amendable to pharmacological activation.

Given the strong protective effect of NS1619 on ROS production, we further investigated its impact on oxidative stress mechanisms relevant to pneumonia, including SOD, catalase, H_2_O_2_ levels, and GSH/GSH ratios^17,18^. Despite NS1619 having no effect on neutrophil-specific MPO secretion, intratracheal (*i.t.*) treatment significantly enhanced antioxidant defenses in BALF cells by rescuing SOD and catalase levels and reducing intracellular H_2_O_2_ concentrations. Notably, while previous studies have shown crosstalk between ROS, MPO/NE, and NETosis pathways^19–21^, our findings suggest that BK activation primarily inhibits ROS pathways. Although CCL-2 and ROS can be produced by various cell types, including immune/inflammatory cells and lung resident cells^4,11,14,23,24^, we also examined whether NS1619 affected markers of neutrophil activation (the predominant cell type in the BALF in our model), such as MPO and NE secretion and NET formation. These findings suggest that either BK activation selectively regulates neutrophil oxidative stress responses but not other neutrophil effector mechanisms, or that the relatively small population of BALF macrophages (only about 8% of BALF cells) plays an important role in BK-mediated regulation of ROS production.

Ion channels, particularly K^+^ channels, play a regulatory role in oxidative stress-related diseases, primarily in cardiovascular^30^ and neurodegenerative disorders^31^. For example, K_ATP_ channels are known to contribute to conditions where oxidative stress is a key factor^32^. One study found that K_ATP_ channel modulators affected morphine-induced antinociception and hepatic oxidative stress, with the K_ATP_ inhibitor glibenclamide reducing hepatic oxidative stress by increasing antioxidants like GSH and GPx, while the K_ATP_ opener diazoxide worsened the condition^33^. The authors speculated that K_ATP_ activation may reduce cell excitability and opioid release in the brain, although the exact mechanism remains unclear. Conversely, morphine-induced oxidative damage is linked to ROS release, which opens K_ATP_ channels^34,35^, highlighting the close relationship between ROS production and ion channel regulation. In contrast, our study in the lung shows that BK channel-induced hyperpolarization reduces oxidative stress, unlike the amplification observed in the brain. This suggests that oxidative stress regulation by K^+^ channels may vary by organ^36,37^.

The exact mechanism by which BK channels regulate ROS production, particularly in lung inflammation, remains unclear. Our study provides the first insights into how BK channel activation can inhibit oxidative stress pathways during pulmonary inflammation by restoring SOD and catalase levels in BALF cells and reducing H_2_O_2_ production (Fig. 3), although other protective mechanisms cannot be ruled out. Based on previous research, one interesting possibility is that BK channels may function as enzymes. This study showed that BK channels possess redox enzyme activity, attributed to their aldo–keto reductase properties, converting aldehydes to alcohols using NADPH as a cofactor^38^. The oxidation of this cofactor, which binds to the β-subunits of BK channels, could potentiate K^+^ currents through Kv1 channels in a voltage-dependent manner^38^, potentially linking BK channel activation to ROS regulation in LPS-induced pneumonia.

Conversely, oxidative stress caused by LPS administration may activate BK channels^39^. In rabbit colonic muscularis, monochloramine (formed by H_2_O_2_ and MPO during inflammation) enhanced BK channel activity, shifting activation to more negative voltages and inducing hyperpolarization^39^, which in our study appears protective against lung injury^13^.. To further explore this pathway, we pharmacologically activated BK channels, which reduced ROS release and restored SOD and catalase levels (Figs. 1 and 3). Additionally, nitric oxide has been shown to interact with BK channels in various cell types, suggesting that nitric oxide may modulate BK activity through redox changes in critical thiol groups^38,40^. This may involve cyclic GMP-dependent protein kinases, as cGMP increases antioxidant function and reduces oxidative cell death^41^, potentially contributing to the protective effects we observed. However, our study did not specifically address the role of cGMP, and further research is needed to explore its relationship with BK activation during oxidative stress.

The other possible mechanism for BK channel activation-mediated regulation of SOD and catalase activities involves intracellular Ca^2+^ levels. Studies show that Ca^2+^ induces conformational changes that regulate SOD and catalase activities in the rabbit urinary bladder^42,43^. Our previous work demonstrated that BK channel activation reduces intracellular Ca^2+^ levels during LPS-induced inflammation^13^, suggesting BK activation may regulate Ca^2+^-sensitive SOD and catalase activities. Additionally, SOD and catalase activities are pH-dependent, with optimal activity at pH 7.0–7.5^44,45^. BK channel-induced Em hyperpolarization influences membrane H^+^ fluxes^46^, and we have previously shown that the human voltage-gated H^+^ channel Hv1 plays a key role in ROS production during LPS-induced inflammation^14^. Em depolarization from NOX2 activation in neutrophils increases H^+^ efflux^47^, and BK channel-induced hyperpolarization may counteract this effect by closing H^+^ channels, reducing H^+^ efflux, and restoring SOD and catalase levels, thereby protecting against oxidative stress^14^. However, this predicted mechanism requires further investigation.

To explore the contributions of resident alveolar cell types in BK channel-mediated regulation of ROS production during LPS-induced inflammation, we examined primary human pulmonary alveolar epithelial and endothelial cells. Our data show that pharmacological BK channel activation reduced LPS-induced ROS production in epithelial cells, whereas endothelial cells did not respond to LPS stimulation with ROS production (Fig. 4). Interestingly, in a previous study using LPS stimulation, we found that NS1619 and NS191504 affect TNF-α- and LPS-induced CCL-2 release predominantly from endothelial but not epithelial cells^25^. Together, these results suggest that in endothelial cells, BK channel activation exerts anti-inflammatory effects by modulating CCL-2 release and, therefore, neutrophil/macrophage migration and activation, while in epithelial cells and BALF immune/inflammatory cells, BK activation reduces LPS-induced ROS production (Figs. 1 and 4). Overall, these data suggest that pharmacological activation of BK channels affects multiple cell types within the alveolar unit, reducing inflammation by limiting cytokine release from endothelial cells and suppressing epithelial and immune/inflammatory cell ROS production (Fig. 5b).

Our in silico model of BK-mediated protection (Fig. 5a) enhances our understanding of how ROS production and cytokine release could be interconnected in vivo. The model predicts that the oxidative stress/ROS and inflammation/cytokine clusters are linked through H_2_O_2_, with key ROS components (catalase, SOD1, SOD2, and the elements copper/zinc) strongly connected to H_2_O_2_. SOD converts superoxide anions to H_2_O_2_, while catalase and GPx convert H_2_O_2_ to water^17,18^. The copper/zinc-SOD catalyzes oxidation through H_2_O_2_ at its active copper site^48^. Although we did not identify the specific type of SOD involved in LPS-induced pneumonia, our model warrants future exploration in this area. In the cytokine cluster, CCL-2, CCL-3, and CXCL-10 are directly linked to H_2_O_2_, which activates chemokine expression through the ERK pathway and transcription factors like Nf-κB and AP-1^49^. Our model shows that BK channels, activated by NS1619 and NS1950, impact the cytokine cluster through CCL-2 and the ROS cluster in two ways: (i) via the cytokine cluster linked to ROS through H_2_O_2_, and (ii) by modulating catalase and SOD activity, as also shown experimentally. This predictive model offers valuable insights into potential connections between BK channels, cytokines, and oxidative stress in LPS-induced pneumonia, guiding future studies to explore these links further.

Study limitations: Although our study shows for the first time the regulatory role of BK channels in LPS-induced pneumonia in vivo, several limitations should be mentioned: (i) While the LPS model is well-established to study gram-negative inflammation, in future studies we need to replicate our exciting findings using live bacteria. (ii) In our model, we administered the first dose of NS1619 simultaneously with LPS. In future studies it will be important to determine whether we can achieve similar protective effects with NS1619 when administered later in the time course of LPS-induced inflammation, which would have therapeutic consequences. (iii) Although the large majority of BALF cells in LPS-infected mice are neutrophils (> 92%) and are thus likely key contributors to our measured endpoints, it is important to not discount potentially significant contributions of other immune/inflammatory cells (e.g. macrophages). ROS, SOD, catalase, H_2_O_2_ and CCL-2 are not immune/inflammatory cell-specific, and in future experiments one could separate neutrophils from macrophages using FCAS or immunomagnetic bead technology to study these cells in isolation. However, for the current study, we first needed to establish that NS1619/NS19504 injections indeed improve the LPS-induced inflammatory environment in the alveolar compartment. (iv) ROS have profound direct cytotoxic and proinflammatory properties, but it will be important to explore whether the observed protective effects of NS1619 and NS19504 could be partially mediated indirectly, e.g. by preventing ROS-mediated activation of other downstream inflammatory signaling cascades such as MAP kinases.

In summary, the results of our study propose pharmacological BK channel activation as a novel and viable strategy to counteract gram-negative pulmonary infections. This approach appears particularly effective in decreasing inflammatory cell infiltration and oxidative stress pathways. Future studies are necessary to further dissect the electrochemical signaling processes underlying BK-mediated protection, and to define the pharmacokinetic and pharmacodynamic profiles of BK channel activators such as NS1619 and NS19504.

## Methods

### Mice

Age (9–12 weeks old)- and sex-matched C57BL/6 J mice were purchased from Jackson Laboratories. Homozygous global BK-KO mice (on C57BL6Jbg background) were a gift from Dr. Meredith^50^. Approval for all experiments was obtained from the University of California Los Angeles Animal Research Committee. All methods were performed in accordance with the University of California Los Angeles Animal Research Committee’s guidelines and regulations. The study was performed in accordance with Animal Research: Reporting In Vivo Experiments (ARRIVE) guidelines. Mouse euthanasia was performed using a terminal dose of ketamine and xylazine, followed by cardiac puncture and lung removal. Survival rates exceeded 97%, with a dropout rate of less than 3%, all of which were attributed to technical or surgical complications. Animals were randomly assigned to the experimental groups in a blinded manner. Investigators remained blinded to the experimental conditions during data analysis.

### Cells

Primary human pulmonary artery endothelial cells (HPAEC) were purchased from Lifeline Cell Technology (Carlsbad, CA, USA) and cultured in an endothelial cell medium (ScienCell, Carlsbad, CA, USA), supplemented with 5% fetal bovine serum (FBS), 1% endothelial cell growth supplement, and 1% antibiotic solution (Penicillin/Streptomycin) (ScienCell, Carlsbad, CA, USA). Primary human alveolar epithelial cells (HPAEpiC) were purchased from ScienCell (Carlsbad, CA, USA) and cultured in a human epithelial cell medium (Cell Biologics, Chicago, IL, USA), supplemented with the epithelial cell medium supplement kit (Cell Biologics, Chicago, IL, USA). Both cell types were used at a passage number < 15. Separate passages from a given lot were considered biological replicates.

### LPS exposure

We injected WT (C57BL/6 J) and BK-KO mice intra-tracheally (*i.t.*) with LPS (10 mg/kg; Cell Signaling, Danvers, MA; *E. coli* serotype O111:B4) or vehicle controls using fiberoptic intubation and a micropipettor under brief inhaled isoflurane anesthesia. Separate groups also received *i.t.* injections of the BK channel activator NS1619 (0.66 mg/kg in 50 µl in sterile PBS; Millipore, Burlington, MA) or NS19504 (1.33 mg/kg in 50 µl in sterile PBS; Alomone labs, Israel), the BK channel blocker Paxilline (1.33 mg/kg in 50 µl in sterile PBS; Alomone labs, Israel), or equimolar vehicle controls, at times 0 and 24 h. We then quantified the degree of lung injury at 48 h. This model resulted in a moderate degree of lung injury with potential for recovery (< 3% mortality), representing a clinically relevant infection.

### Bronchoalveolar lavage fluid (BALF) collection, total and differential cell count quantification

A tracheostomy was performed with an 18 g steel catheter under ketamine/xylazine anesthesia. BALF was collected in 1 ml cold PBS/0.6 mM EDTA for total and differential cell count determination, and in 1 ml PBS/1% BSA for cytokine quantification. All samples were immediately placed on ice. Total BAL cell counts were performed using a Bright Line Counting Chamber (Hausser Scientific, Horsham, PA) and differential cell counts using a Diff-Quick stain (Thermo Fisher, Waltham, MA). BALF supernatants and cell pellets were separated by centrifugation (2500 rpm × 10 min at 4 °C)^4^.

### Lung histology and lung injury score (LIS) determination

Lung tissue was harvested and processed as described previously^11^. Briefly, the heart and lungs were removed *en bloc* and gently retrograde perfused with 10 mL of ice-cold PBS to remove red blood cells from the vasculature. The lungs were then fixed in 4% formalin, and paraffin-embedded sections were cut into 4-µm thick tissue slices using a Microtome. Lung injury scores (LIS) were determined by an investigator blinded to the experimental conditions on hematoxylin–eosin (H&E)-stained lung sections using our previously published 3-criteria LIS^11^: (1) interstitial edema, (2) inflammatory cell infiltrate, and (3) parenchymal, peribronchial, and perivascular hemorrhage. Each of the three criteria was assigned a score between 0 and 3, with “0” representing no injury, “1” representing mild injury, “2” representing moderate injury, and “3” representing severe injury. Four random high-power fields per slide were scored under × 10 and × 20 magnification and averaged for each criterion. The derived composite histological LIS represents the sum of the three parameters for a given condition. All histological analyses were performed using a Nikon E800 microscope and AmScope camera with included software.

### CCL-2 cytokine measurements

CCL-2, CCL-3 and CXCL-10 levels in BALF were quantified using species-specific ELISAs (BD Biosciences, San Jose, CA) following the manufacturer’s instructions.

### ROS measurements

Cytosolic ROS levels were assessed using a DCFDA/H2DCFDA Cellular ROS Assay Kit (Abcam, Waltham, MA) and mitochondrial ROS levels using a MitoSOX Red mitochondrial superoxide indicator (Thermo Fisher, Waltham, MA). H_2_O_2_ levels were quantified using an Amplex™ Red Hydrogen Peroxide/Peroxidase Assay Kit (Fisher Scientific, Hampton, NH).

### Catalase, superoxide dismutase (SOD) and glutathione peroxidase (GPx) activity measurements

Catalase (Catalase Activity Assay kit; Abcam, Waltham, MA) and SOD (SOD ELISA kit, Abcam, Waltham, MA) activity we measured at 450 nm using a fluorescence plate reader (Molecular Devices, San Jose, CA). To measure GPx activity (GSH/GSSG Ratio Detection Assay Kit, Abcam, Waltham, MA) we quantified the ratio of reduced glutathione (GSH) to oxidized GSH (GSSG).

### Neutrophil myeloperoxidase (MPO), neutrophil elastase (NE), and neutrophil extracellular traps (NETosis) measurements

MPO and NE protein levels were assessed using a Mouse Myeloperoxidase DuoSet Elisa kit (R&D systems, Minneapolis, MN) and a Mouse Neutrophil Elastase DuoSet Elisa kit (R&D systems, Minneapolis, MN), respectively. NETosis was quantified using MPO-specific or NE-specific capture antibodies, followed by MPO-DNA or NE-DNA antibody detection (Cell Death Detection ELISA, Roche, Basel, Switzerland).

### Prediction in silico model using functional enrichment analysis (STITCH)

To predict the BK-channel mediated network underlying the interactions between BK channels, CCL-2, CCL-3 and CXCL-10, SOD and catalase, we input these variables into a search tool for the retrieval of interacting genes/proteins/drugs and chemicals (STITCH; http://stitch.embl.de/) following our previously described strategy^5^.

### Statistics

Data are expressed as mean ± SEM, or median with min. and max. values. Comparisons were performed using Mann–Whitney U or Student t-tests (GraphPad 8.3, San Diego, CA), or two-ways ANOVA; Exact sample sizes are depicted as individual data points in each graph, with ranges reported in the figure legends. A *p* value of ≤ 0.05 was considered statistically significant and biologically relevant, and is reported for each data set comparison. All data points were included, except for outliers that fell outside of 1 standard deviation.

## Data Availability

All data generated or analyzed during this study are included in this published article.
